# Viability of homologous and heterologous subcutaneous transplantation of fresh germ cells in rabbits

**DOI:** 10.5935/1518-0557.20170019

**Published:** 2017

**Authors:** Paula M.A. Cerialle, Carlos G. Almodin, Moacir R. M. Radaelli, Vânia C. Minguetti-Câmara, Michelle C. Souza, Carlos A.M. Oliveira, Antonio J. Gonçalves

**Affiliations:** 1 Materbaby - Human Reproduction and Genetics, Maringá, Brazil; 2 Ingá University Center- UNINGA, Maringá, Brazil; 3 Núcleo Santista - Human Reproduction, Santos, Brazil; 4 Surgery Department, Medical Sciences School at the Santa Casa de São Paulo Hospital, São Paulo, Brazil

**Keywords:** Germ cells, transplantation, heterologous, rabbits

## Abstract

**Objective:**

This study aimed to compare heterologous to homologous transplantation of fresh ovarian germ cells in rabbits.

**Methods:**

Twelve female white New Zealand rabbits (Oryctolagus cuniculus) were randomly numbered and submitted to bilateral oophorectomies. The ovaries from the six odd-numbered rabbits were dissected and cortical germinal tissue was digested in collagenase type 1 to obtain six solutions containing stromal and germ cells, which were injected in the abdominal region of the odd-numbered rabbits themselves (homologous transplantation) and of the even-numbered rabbits (heterologous transplantation) off immunosuppression. Sixty days after transplantation, the tissue around the transplanted region was excised, processed and sent to histological analysis with hematoxylin-eosin staining and Bcl-2 immunohistochemistry to verify the presence and viability of the transplanted cells.

**Results:**

The analyzed specimens contained ovarian stroma, while follicular cells were found in 66.6% of the homologous and in 60% of the heterologous transplant specimens. Mild inflammatory reaction was observed in all heterologous specimens, and in only one (16.7%) of the homologous specimens. However, this inflammatory reaction was not so intense as to cause the death of the implanted cells. Except for the specimens from rabbits 7 and 8, all specimens were stained for Bcl-2, indicating that most of them were viable.

**Conclusions:**

The results of this study supported the viability of heterologous transplantation of fresh ovarian germ cells. However, more studies are required to further our understanding and improve the germ cell separation technique.

## INTRODUCTION

In recent decades many women have opted to have children at a later age and, as a result, the number of infertile women has increased significantly. Moreover, one in every 250 women under the age of 35 suffer from primary ovarian failure (POF) and the consequences of this event (^Aubard *et al.*, 1998^). In addition to fertility issues, women with POF also suffer from physical effects of hypoestrogenism such as vulvovaginal atrophy, decalcification, cardiovascular disease, and psycho-emotional disorders (^De Vos *et al.*, 2010^; ^Wolff *et al.*, 2013^). Although several studies have been conducted to find appropriate solutions for this group of women (^Bhavnani & Stanczyk, 2014^; ^Gaowa *et al.*, 2015^), medicine has not been able to find an effective solution to these problems.

Heterologous insemination, and the donation and reception of oocytes from different women have been successfully employed for more than two decades in routine infertility treatments in human reproduction centers all over the world (^Cobo *et al.*, 2015^). Although oocyte donation is a possible solution to infertility, the only solution available at this point for the physical changes resulting from estrogen deprivation is hormone replacement therapy (^Pinto Marín *et al.*, 2011^).

In an attempt to deal with infertility and the hormonal problems caused by POF, animal model studies have focused on the recovery of ovarian function by freezing ovarian germinal tissue for later reimplantation (^Almodin *et al.*, 2004a^, ^2004b^). Based on these studies, the first live birth of a child after orthotopic autotransplantation of cryopreserved ovarian tissue was reported in 2004 (^Donnez *et al.*, 2004^). Restoration of ovarian function and fertility, followed by the birth of a healthy baby, were also reported after the combined orthotopic and heterotopic transplantation of cryopreserved ovarian tissue in a 31-year-old woman previously treated for Hodgkin's disease (^Demeestere *et al.*, 2007^). A recent review reported the outcomes of 60 orthotopic reimplantations of cryopreserved ovarian tissue performed by three groups of researchers, as well as 24 live births (^Donnez *et al.*, 2013^).

Despite the success achieved with homologous transplantation of ovarian germinal tissue, this procedure is not an option for many women diagnosed with POF who, for different reasons, do not possess cryopreserved ovarian tissue. In theory, the transplantation of germinal tissue received from other women might benefit this group of patients. A recent study reported on ten monozygotic twins discordant for POF offered ovarian transplantation with fresh and cryopreserved tissue with successful pregnancies, with the oldest graft functioning for 36 months (^Silber *et al.*, 2008^). These results suggest that heterologous transplantation may be viable.

Considering that rejection is not an issue in the transplantation of germ cells such as oocytes and sperm, it would seem logical to infer that the germ cells present in the ovary (stroma and follicles) would behave in a similar fashion. However, in an animal model study conducted with hens, the authors demonstrated that the fibroblastlike cells in the theca layer of primary follicles were positive for molecular histocompatibility complex class II (MHC-II), which is responsible for immunoreaction in the ovaries (^Barua & Yoshimura, 1999^). Thus, if ovarian germ cells were separated from the MHC-II-positive fibroblasts present in the surrounding tissue, the probability of the germ cells being rejected in the host site would be minimized, enabling the heterologous transplantation of these cells.

Therefore, this study aimed to evaluate the viability of subcutaneous heterologous transplantation of fresh germ cells in rabbits versus homologous transplantation.

## MATERIALS AND METHODS

### Ethics committee approval

The Institutional Animal Care and Use Committee (IACUC) at Ingá University Center (UNINGA), Maringá, Brazil, approved this animal model study. The procedures performed in this study were carried out at the Animal Surgery facilities of UNINGA with the technical support of Materbaby - Human Reproduction and Genetics between February 2016 and May 2016. The procedures were carried out in accordance with the guidelines established by the Brazilian College of Animal Experimentation (COBEA).

### Animals

The study population consisted of twelve female white New Zealand rabbits (*Oryctolagus cuniculus*) obtained from Central Animal Laboratory of the Medical School of the University of São Paulo, São Paulo, Brazil. With ages ranging between three and six months and weighing between 2.5 and 4kg, the rabbits were specifically raised for research purposes. The number of subjects used in this experiment was based on previous animal studies (^Almodin *et al.*, 2004^, ^2004^). None of the rabbits had physical anomalies, ectopic ovaries, ovarian malformations, or tumors, which might adversely impact study outcomes.

Each of the rabbits was placed in a standard individually identified polypropylene cage (50x30x50cm) in the vivarium at UNINGA. They were kept at constant temperature (around 25°C) on a 12-hour light/12-hour dark cycle for 40 days before the procedures were initiated to ensure they were fit for the experiment. The rabbits were given water and food proper for the species (Nuvilab CR1, Nuvital, Colombo, PR) ad libitum during their stay at the vivarium.

### Surgical Procedures

The twelve rabbits were randomly numbered from 1 to 12 and identified by a tattoo on the inner portion of the right ear. Odd-numbered rabbits were assigned to the homologous transplantation group, while even-numbered rabbits were placed in the heterologous transplantation group.

The subjects were submitted to bilateral oophorectomies in pairs (1 and 2 etc.). On the day of surgery, food and water was withdrawn two hours before the procedure, and pre-anesthesia with acepromazine 0.2% (0.1mg/kg) was performed 30 min before surgery to mitigate stress. Then, the rabbits were taken to the operating room, where they were administered general anesthesia with xylazine 2% (10mg/kg) and ketamine 10% (75mg/kg) (Bayer, Leverkusen, Germany). Oxygen therapy (3l/min) was maintained throughout the surgical procedure. The animals were considered anesthetized when they did not respond to handling and to susceptibility tests. The subjects were anesthetized by a veterinary physician specialized in animal anesthesia.

Both ovaries were surgically removed and placed in Petri dishes containing cold buffer solution with glucose and pyruvate supplemented with 10% synthetic serum - PBS (Dubecco's Ingamed^®^, Maringá, Brazil); the specimens were immediately sent to the reproduction laboratory. At the end of the procedure, the subjects were administered 0.2mg/kg of meloxicam (0.2mg/kg) as a long-term anti-inflammatory agent (24 hours).

### Germ Cell Solution

Once in the reproduction laboratory, the ovaries were longitudinally dissected with a scalpel; the medullar part was discarded, while the cortical layer was sectioned into small pieces (approximately 3mm^2^). The germinal tissue fragments from the odd-numbered rabbits were digested in collagenase type 1, following a protocol previously described with some modifications (^Osterholzer *et al.*, 1985^): i) the ovarian tissue fragments were washed five times with PBS to remove blood and debris; ii) then the specimens were placed in 15-ml centrifuge tubes with collagenase type 1 300IU/ml (Sigma- Aldrich, St. Louis, USA) diluted in PBS; iii) the solution was gently homogenized for 45 minutes to allow tissue digestion and the separation of germ cells; iv) the solution was centrifuged at 200G for 10 minutes; v) the supernatant was removed and the resulting precipitate was washed with PBS twice to remove residual collagenase; and vi) the precipitate was then diluted in 1.0ml of PBS. Six solutions were prepared, one for each odd-numbered rabbit.

### Germ Cell Transplantation

Two insulin syringes equipped with 18G needles (1.2mm diameter) were prepared with 0.5ml of solution containing germ cells; the contents of one were injected in the donator odd-numbered rabbit (homologous transplantation) and the other on the subsequent even-numbered rabbit (heterologous transplantation). Before the injection, a sterile stainless steel marker 0.7mm in diameter and 10mm long was inserted into the distal end of the needle. The transplantations were performed as part of the same surgical procedure, approximately 75 minutes after the oophorectomy had been performed. The solution containing germ cells was injected subcutaneously, together with the metal marker, in the abdomen of the rabbits two centimeters below the bottom left nipple. After transplantation, the rabbits were returned to the vivarium and kept in the same room under the same conditions with ad libitum supply of food and water until the excisional biopsy.

### Transplanted Tissue Analysis

Sixty days after transplantation, an excisional biopsy of approximately 2cm in diameter was performed in each of the subjects in the subcutaneous tissue around the metallic marker; the specimens were sent to histological assessment and immunohistochemistry testing.

The slides stained with hematoxylin-eosin provided data for the histological assessment. The parameters studied were the presence of ovarian stroma, follicular cells, and signs of necrosis and inflammation.

Immunohistochemistry tests were performed with a marker for the oncogenic protein that inhibits apoptosis (Bcl-2), which is found within the mitochondrial membrane, endoplasmic reticulum and nuclear envelope. Sections were incubated with primary antibodies (clone 124 - DAKO Corp., Carpintería, CA) prepared in a previously optimized Bcl-2 solution. In order to allow the visualization of the reaction, the sections were treated with a chromogenic substrate (diaminobenzidine 60mg % in PBS with 1.5ml of hydrogen peroxide 20vol) for five minutes at 37ºC. The sections were counterstained with Harris hematoxylin (HHS128 - Sigma-Aldrich, St. Louis, USA), dehydrated, and mounted on the slides with Entellan medium (Sigma-Aldrich, St. Louis, USA). The slides stained with the Bcl2 anti-apoptotic marker were used to demonstrate germ cell viability. Positive reactions produced a sepia color (chromogenic substrate). The qualitative criteria for categorizing the immunohistochemical expression of Bcl-2 were based on the parameters used for the determination of HER-2 scores in the HercepTest^TM^ kit (DAKO Corp., Carpinteria, CA). Table 1 shows the scores assigned to immunostaining.

**Table 1:** Bcl-2 staining scores

Immunostaining standard	Score	Immunoreactivity
Weak cytoplasmic or nuclear staining	1+	Negative
Strong cytoplasmic or nuclear staining	2+	Mildly positive
Strong nuclear and cytoplasmic staining	3+	Strongly positive

### Statistical Analysis

No statistical analysis was conducted. The results of histological and immunohistochemical evaluations were analyzed descriptively.

## RESULTS

Rabbit number 2 (heterologous group) died four days before the biopsy for unknown reasons, and was excluded from the study. The remaining 11 rabbits recovered from surgery uneventfully.

### Fresh Tissue And Solutions

**Figure 1:**
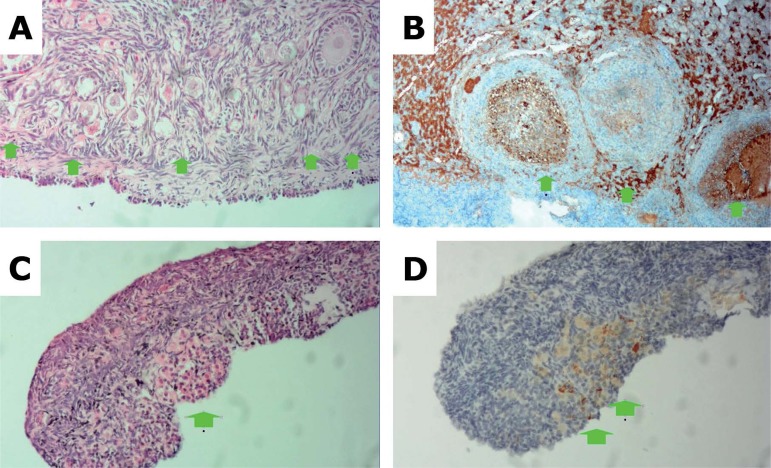
(A) Fresh ovarian germinal tissue specimen stained by HE (rabbit 5) showing multiple follicles. (B) Immunohistochemistry staining of fresh ovarian germinal tissue specimen showing several follicles (arrows) with strong Bcl-2 staining in sepia color (3+). (C) HE-stained germ cell solution (solution 3) revealing the presence of ovarian tissue (elongated stromal cells) and the outline of a follicle (arrow). (D) Immunohistochemistry staining of germ cell solution with weak sepia Bcl-2 staining (2+).

Histological analysis of the fresh biopsy specimens collected from the subjects revealed the presence of stroma and follicles in different stages of development, while immunohistochemistry showed strong Bcl-2 staining (3+), indicating that the rabbits were at reproductive age. Histology tests showed that the six solutions originating from the fresh germinal tissue of the odd-numbered rabbits contained a lower number of germ cells (stromal cells and follicles). Immunohistochemical assessment of all solutions also revealed decreased Bcl-2 staining. While the score for fresh ovarian tissue was 3+ (strongly positive), the score for the solutions was 2+ (weakly positive). With the exception of solution 4 (rabbit 7), which obtained score 1+ (negative), the other five solutions were deemed to contain viable germ cells (Figure 1).

### Transplanted Tissue

**Figure 2:**
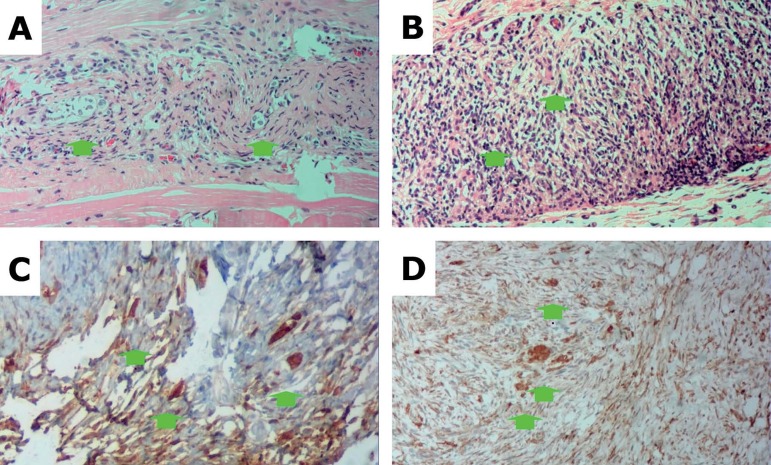
(A) Excisional biopsy specimen from a rabbit given a homologous graft (Rabbit 3) stained with HE showing ovarian tissue containing stroma and a few primordial follicles (arrows) among the soft tissue, absence of necrosis or inflammatory cells. (B) Excisional biopsy specimen from a rabbit given a heterologous graft (rabbit 4) stained with HE, showing ovarian tissue containing stroma and a few primordial follicles among the soft tissue, absence of necrosis, and presence of inflammatory cells. (C) Excisional biopsy specimen from a rabbit given a homologous graft (rabbit 3) with Bcl-2 staining, showing strong sepia marking (3+) in the cell cytoplasm. (D) Excisional biopsy specimen from a rabbit given a heterologous graft (rabbit 4) with Bcl-2 staining showing strong marking (3+) in sepia color in the cell cytoplasm.

Table 2 shows the histology and immunohistochemistry test results for transplanted tissue. Ovarian stroma was present in four (80%) of the five rabbits in the heterologous group, and in five (83.3%) of the six rabbits in the homologous group. The two rabbits that had no ovarian stroma (rabbits 7 and 8) had been injected with solution 4. Follicular cells were present in three (60.0%) of the five rabbits in the heterologous group, and in four (66.6%) of six rabbits in the homologous group. Three rabbits in the heterologous group (rabbits 6, 10 and 12), and two in the homologous groups (9 and 11) had 2+ scores in Bcl-2 staining. One of the rabbits given a heterologous graft (rabbit 4), and three of the rabbits given homologous graft (1, 3 and 5), had 3+ scores in Bcl-2 staining. All rabbits (100%) in the heterologous group, and only one subject (rabbit number 5) in the homologous group, had inflammatory cells in their excisional biopsy specimens (Figure 2).

**Table 2:** Comparison of heterologous and homologous transplantation groups for presence of ovarian stroma, follicular cells, Bcl-2 marking, and inflammatory cells in the 11 rabbits evaluated

Variables	Groups				
	HETEROLOGOUS (n=5)		HOMOLOGOUS (n=6)	
	N	%	N	%
**Ovarian stroma**				
Absent	1	20	1	16.7
Present	4	80	5	83.3
**Follicular cells**				
Absent	2	40	2	33.33
Present	3	60	4	66.66
**BCL2 marking**				
1+	1	20	1	16.7
2+	3	60	2	33.3
3+	1	20	3	50
**Inflammatory cells**				
Absent	0	0	5	83.3
Present	5	100	1	16.7

## DISCUSSION

This study compared heterologous to homologous transplantation of germ cells in the abdominal region of white New Zealand rabbits. To our knowledge, this is the first study to attempt the transplantation of germ cells from one animal to another (heterologous transplantation) without the use of immunosuppressants. The study demonstrated promising results, with the presence of stromal and follicular cells and the absence of apoptotic cells in both groups of subjects analyzed.

The enzymatic digestion of germinal tissue was performed to separate germ cells from the surrounding tissue in an attempt to eliminate the MCH-II-positive cells found in connective tissue and minimize the effects of rejection. The injection of germ cells only (follicles and stroma) was expected to facilitate angiogenesis and neovascularization at the receptor site. Previous research showed that the outcomes of heterotopic or orthotopic transplantation of ovarian tissue in mice were significantly impaired when neovascularization did not occur within 48 hours of transplantation (^Demeestere *et al.*, 2009^). The authors also showed that stromal integrity is essential for the process of neovascularization and survival of follicular tissue. Primordial follicles can withstand ischemia for four hours, but stromal cells surrounding follicles are more sensitive to ischemia than primordial follicles. This finding has been associated with dramatic decreases in transplanted tissue viability, which might undergo fibrosis (^Demeestere *et al.*, 2009^).

To avoid ischemia-related issues, in the present study the solution containing germ cells was injected in the homologous and heterologous subjects during the same surgical session, about 75 minutes after the removal of the ovaries. The small size of the ovaries and the small amount of solution obtained required that homologous/heterologous transplantations be carried out in pairs. The 60 days between transplantation and collection of biopsy specimens provided ample time not only to assess the viability of the implanted tissue, but also to look into the possible effects of transplantation on the health status of the subjects. Although one of the rabbits given a heterologous implant (rabbit 2) died just before biopsy for no apparent reason, all the other subjects recovered uneventfully.

The protocol used for the production of the solution containing germ cells was based on a previous study (^Osterholzer *et al.*, 1985^). However, modifications were made in an attempt to minimize the possible damage caused to germ cells. A buffered medium (PBS) with a more stable pH was used to rinse the tissue and prepare the solutions. Germinal tissue specimens (before digestion) and germ cells (after digestion) underwent several rinsing steps to remove possible impurities and residual collagenase, respectively, which otherwise might have compromised our outcomes. Exposure time to collagenase was also decreased from 60 to 45 min to reduce the risk of damage to the follicles.

A recent study revealed that the ovarian extracellular matrix between the follicles can be digested by collagenase type 1. However, the collagen present within the follicles can also be degraded and damage follicle integrity (^He & Toth, 2016^). This could explain the fact that, although the ovarian tissue of rabbit 7 provided a follicle-rich germinal tissue specimen, the solution produced from this tissue (solution 4) had a negative score (1+) for Bcl-2 staining. This finding indicated that the follicles might have been damaged during digestion, and suggested that more studies should be performed before an ideal protocol is developed for the separation of ovarian germ cells.

As expected, inflammatory reaction was more commonly found in heterologous than in homologous grafts. Inflammatory cells were observed in only one subject given a homologous graft (rabbit 5), most likely due to trauma caused by the transplantation procedure. However, the inflammatory reaction observed in the present study was not so intense as to promote the death of germ cells, and no signs of rejection were observed in the transplanted tissue.

The design used in this study allowed, with a good degree of reliability, the production of tissue specimens containing germ cells. The use of a sterile marker injected with the solution made it possible. The findings, and particularly the data from rabbits in the homologous graft group, seem to indicate that the marker had no impact on the outcomes. This method has been successfully used in previous animal studies, demonstrating the benefits of using the marker (^Almodin *et al.*, 2004^, ^2004^).

The transplantation of germ cells was performed in a heterotopic site to facilitate tissue collection and analysis. However, this procedure carries some inherent limitations. It was recently reported that one of the main reasons for decreased graft viability is the unsuitability of the heterotopic environment for the neovascularization process, due to changes in temperature, pressure, lack of space for follicular growth, as well as the presence of cytokines, and angiogenic and hormonal factors.

Despite the preliminary nature of the results reported in this study, its encouraging findings support the organization of future studies on the development of solutions for selected POF patients. Orthotopic transplantation, associated with an adequate protocol for the isolation of ovarian germ cells, may offer more promising results. However, further research is required to better understand the antigenic factors involved in the transplantation of germ cells, and to establish an optimal ovarian germ cell separation protocol.

## CONCLUSIONS

Given the conditions of the present study, heterologous and homologous transplantation of germ cells in rabbits had similar outcomes and were proven viable procedures.

## References

[r1] Almodin CG, Minguetti-Câmara VC, Meister H, Ceschin AP, Kriger E, Ferreira JO (2004a). Recovery ofnatural fertility after grafting of cryopreserved
germinative tissue in ewes subjected to radiotherapy. Fertil Steril.

[r2] Almodin CG, Minguetti-Câmara VC, Meister H, Ferreira JO, Franco RL, Cavalcante AA, Radaelli MR, Bahls AS, Moron AF, Murta CG (2004b). Recovery of fertility after grafting of cryopreserved germinative
tissue in female rabbits following radiotherapy. Hum Reprod.

[r3] Aubard Y Newton H, SchefferG Gosden R (1998). Conservation of the follicular population in irradiated rats by
the cryopreservation and orthotopic autografting of ovarian
tissue. Eur J Obstet Gynecol Reprod Biol.

[r4] Barua A, Yoshimura Y (1999). Immunolocalization of MHC-II + cells in the ovary of immature,
young laying and old laying hens Gallus domesticus. J Reprod Fertil.

[r5] Bhavnani BR, Stanczyk FZ (2014). Pharmacology of conjugated equine estrogens: efficacy, safety and
mechanism of action. J Steroid Biochem Mol Biol.

[r6] Cobo A, Garrido N, Pellicer A, Remohí J (2015). Six years' experience in ovum donation using vitrified oocytes:
report of cumulative outcomes, impact of storage time, and development of a
predictive model for oocyte survival rate. Fertil Steril.

[r7] De Vos M, Devroey P, Fauser BC (2010). Primary ovarian insufficiency. Lancet.

[r8] Demeestere I, Simon P, Emiliani S, Delbaere A, Englert Y (2007). Fertility preservation: successful transplantation of
cryopreserved ovarian tissue in a young patient previously treated for
Hodgkin's disease. Oncologist.

[r9] Demeestere I, Simon P, Emiliani S, Delbaere A, Englert Y (2009). Orthotopic and heterotopic ovarian tissue
transplantation. Hum Reprod Update.

[r10] Donnez J, Dolmans MM, Demylle D, Jadoul P, Pirard C, Squifflet J, Martinez-Madrid B, van Langendonckt A (2004). Live birth after orthotopic transplantation of cryopreserved
ovarian tissue. Lancet.

[r11] Donnez J, Dolmans MM, Pellicer A, Diaz-Garcia C, Sanchez Serrano M, Schmidt KT, Ernst E, Luyckx V, Andersen CY (2013). Restoration of ovarian activity and pregnancy after
transplantation of cryopreserved ovarian tissue: a review of 60 cases of
reimplantation. Fertil Steril.

[r12] Gaowa S, Sun AJ, Jiang Y, He FW, Zheng TP, Wang YP (2015). Ultrasonographic observation of the breast in early
postmenopausal women during therapy with Cimicifuga foetida extract and
sequential therapy with estrogen and progestin. Chin Med J (Engl).

[r13] He X, Toth TL (2016). In vitro culture of ovarian follicles from
Peromyscus. Semin Cell Dev Biol.

[r14] Osterholzer HO, Streibel EJ, Nicosia SV (1985). Growth effects of protein hormones on cultured rabbit ovarian
surface epithelial cells. Biol Reprod.

[r15] Pinto Marín A, Ballesteros García AI, Izarzugaza Perón Y, Mansó Sánchez L, López-Tarruella Cobo S, Zamora Auñón P (2011). Adjuvant hormonal therapy in perimenopausal
patients. Adv Ther.

[r16] Silber SJ, DeRosa M, Pineda J, Lenahan K, Grenia D, Gorman K, Gosden RG (2008). A series of monozygotic twins discordant for ovarian failure:
ovary transplantation (cortical versus microvascular) and
cryopreservation. Hum Reprod.

[r17] Wolff EF, He Y, Black DM, Brinton EA, Budoff MJ, Cedars MI, Hodis HN, Lobo RA, Manson JE, Merriam GR, Miller VM, Naftolin F, Pal L, Santoro N, Zhang H, Harman SM, Taylor HS (2013). Self-reported menopausal symptoms, coronary artery calcification,
and carotid intima-media thickness in recently menopausal women screened for
the Kronos early estrogen prevention study (KEEPS). Fertil Steril.

